# Characteristics of Movement Disorders in Patients with Autoimmune GFAP Astrocytopathy

**DOI:** 10.3390/brainsci12040462

**Published:** 2022-03-29

**Authors:** Akio Kimura, Akira Takekoshi, Takayoshi Shimohata

**Affiliations:** Department of Neurology, Gifu University Graduate School of Medicine, 1-1 Yanagido, Gifu 501-1194, Japan; takekosi@gifu-u.ac.jp (A.T.); shimohata@gmail.com (T.S.)

**Keywords:** astrocytopathy, ataxia, glial fibrillary acidic protein (GFAP), movement disorder, myoclonus, tremor

## Abstract

Autoimmune glial fibrillary acidic protein (GFAP) astrocytopathy (GFAP-A) is a type of autoimmune corticosteroid-responsive meningoencephalitis that occurs with or without myelitis. Movement disorders have been reported in GFAP-A patients but have not been characterized. In this study, we examined the characteristics of movement disorders in GFAP-A patients. We retrospectively reviewed clinical data from 87 consecutive patients with GFAP-A attending Gifu University Hospital in Japan. We compared the demographics, clinical features, cerebrospinal fluid characteristics, and neuroimaging findings from patients with and without movement disorders. Seventy-four patients (85%) had movement disorders, including ataxia (49%), tremor (45%), myoclonus (37%), dyskinesia (2%), opsoclonus (2%), rigidity (2%), myokymia (1%), and choreoathetosis (1%). GFAP-A patients with movement disorders were significantly older than those without. Movement disorders are therefore common in GFAP-A patients, and the main types of movement disorders observed in this population were ataxia, tremor, and myoclonus. These abnormal movements can serve as clinical features that facilitate the early diagnosis of GFAP-A. Elderly GFAP-A patients are more likely to have movement disorder complications than younger patients.

## 1. Introduction

Autoimmune glial fibrillary acidic protein (GFAP) astrocytopathy (GFAP-A) is a type of autoimmune corticosteroid-responsive meningoencephalitis that occurs with or without myelitis [1,2]. The detection of immunoglobulin G (IgG) against GFAP (GFAP-IgG) in cerebrospinal fluid (CSF) is a biomarker of GFAP-A. GFAP-A was first described in 16 patients [1], and since then, it has been documented in several countries, including the USA, China, Italy, the UK, France, and Japan [3,4,5,6,7,8]. Patients generally present with headache and fever, followed by disturbed consciousness and signs of meningeal irritation [2,8]. Blurred vision related to optic disc edema has also been observed [2]. Some GFAP-A patients have a neoplasm such as ovarian teratoma [2,9]. Brain linear perivascular radial gadolinium enhancement (LPRGE) patterns, an imaging hallmark of GFAP-A, are noted in about half of all patients [2,7,8]. A neuropathological study reported inflammatory infiltrates, including T, B, and plasma cells surrounding blood vessels [4]. Although the pathophysiological mechanisms of GFAP-A have not yet been elucidated, GFAP-specific CD8^+^ T cells are likely effectors of this disorder [4,10,11]. GFAP-A is usually corticosteroid-responsive in the acute stage [2,7,8]. However, some patients are prone to relapse or even death [12]. Early diagnosis and initiation of corticosteroid therapies are important for patient prognoses [13].

Previous reports have addressed the movement disorders associated with GFAP-A [14,15,16]. However, most reports are case reports or studies with small sample populations. Therefore, the characteristics of movement disorders in GFAP-A patients remain to be elucidated. Here, we describe the characteristics of movement disorders in GFAP-A patients and consider clinical features that may facilitate the early diagnosis of this condition. 

## 2. Patients and Methods

### 2.1. Patients

We enrolled 492 Japanese patients with inflammatory central nervous system diseases to examine CSF GFAP-IgG in this population. Of the participants, 277 had been admitted to Gifu University Hospital between May 2002 and December 2021. The other 215 participants submitted CSF samples for GFAP-IgG evaluation to Gifu University Graduate School of Medicine between January 2019 and December 2021. We retrospectively reviewed the clinical data from all patients with CSF GFAP-IgG. Clinical information was available via the clinical records of Gifu University Hospital and from communication with referring physicians. The referring neurologists were asked to note the presence and type of movement disorder during the clinical course. Written informed consent was obtained from all patients before the investigation commenced. This study was approved by the institutional review board of Gifu University Graduate School of Medicine, Gifu, Japan (Protocol 27-43).

### 2.2. Measurement of CSF GFAP-IgG

GFAP-IgG was detected in CSF samples via a transfected cell-based assay and a tissue-based immunofluorescence assay, according to a previous report [2,8]. 

### 2.3. Statistical Analysis

Data are reported as the median and range, or the number and percentage. Data analysis was performed using statistical software (Excel-Toukei 2012, Social Survey Research Information; Tokyo, Japan). The Shapiro–Wilk test determined that the data were nonparametrically distributed; therefore, differences in statistical significance between patient groups were calculated using the Mann–Whitney U Test for continuous variables and Fisher’s exact test for categorical variables, as appropriate. A *p*-value ≤ 0.05 was considered statistically significant.

## 3. Results

### 3.1. Demographics, Clinical Features, CSF Characteristics, and Neuroimaging in Autoimmune GFAP-A Patients

Both the transfected cell-based assay and the tissue-based immunofluorescence assay revealed GFAP-IgG in CSF samples from 109 of the 492 patients tested. Among these 109 GFAP-A patients, 22 were excluded because sufficient clinical data were not available. Thus, we evaluated clinical information from 87 GFAP-A patients. The demographics, clinical features, CSF characteristics, and neuroimaging findings from the 87 GFAP-A patients are shown in Table 1. The median age of the 87 patients was 51 years (range: 5–83), and 56 (64%) were male. Fourteen patients (16%) had tumors: ovarian teratoma in five patients, breast cancer in three patients, and one patient each for pituitary adenoma, acoustic tumor, thyroid follicular adenoma, hepatic teratoma, colon cancer, and rectum cancer. Two patients had autoimmune diseases: one had atopic dermatitis and the other had rheumatoid arthritis. The median period from onset to admission was 12 days (range: 1–1054). 

Seventy-four of the 87 GFAP-A patients (85%) had a movement disorder, and this was the most common clinical finding in this patient group. The other common clinical findings were urinary dysfunction (67/87 (77%)), altered consciousness (66/87 (76%)), nuchal rigidity and/or Kernig’s sign (56/87 (64%)), fever (54/85 (64%)), and hyperreflexia (50/86 (58%)). The least common clinical findings were respiratory failure (19/86 (22%)) and convulsion (17/87 (20%)).

The CSF examination showed mononuclear cell-predominant pleocytosis and elevated protein concentrations in almost all of the patients. The median cell count was 79 cells/μL (range: 4–472), and the median protein concentration was 146 mg/dL (range: 30–320). Forty-three out of 61patients (70%) had oligoclonal IgG bands. 

Coexisting neuronal autoantibodies were anti-N-methyl-D-aspartate (NMDAR) (2/42 (5%)), anti-myelin oligodendrocyte glycoprotein (MOG) (2/57 (4%)), and anti-glutamic acid decarboxylase (GAD) antibodies (2/8 (25%)). None of the 66 patients had anti-aquaporin 4 (AQP4) antibodies. 

**Figure 1:**
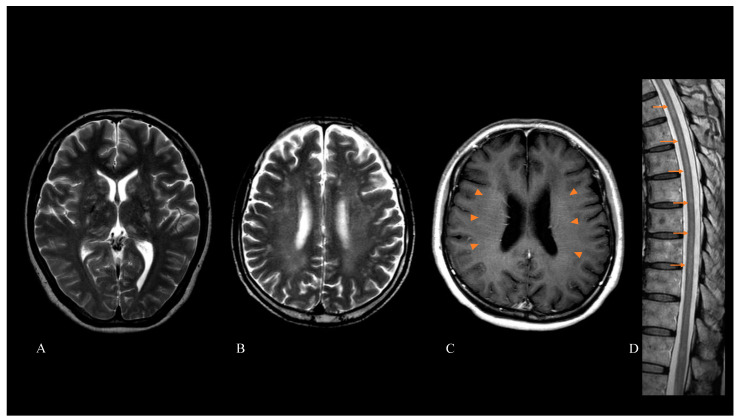
Brain and spinal cord magnetic resonance imaging (MRI) of patients with autoimmune glial fibrillary acidic protein (GFAP) astrocytopathy. Abnormal hyperintensity lesions on T2-weighted imaging were observed in the basal ganglia, thalamus (**A**), and cerebral white matter (**B**). Gadolinium-enhanced brain MRI showed a linear perivascular radial enhancement pattern (arrowheads) (**C**). Spinal cord MRI showed longitudinally extensive T2-hyperintensity lesions (arrow) (**D**).

Brain magnetic resonance imaging (MRI) revealed that 76 out of 86 patients (88%) had abnormal hyperintensity lesions on T2-weighted and fluid-attenuated inversion recovery (FLAIR) images. The hyperintensity lesions were in white matter (49/84 (58%)), the basal ganglia (36/84 (43%)), and cerebellum (5/84 (6%)). When we conducted gadolinium-enhanced brain MRI, 53 out of 78 patients (68%) had abnormal enhancement lesions. Forty-two out of 78 patients (54%) had LPRGE patterns in the cerebral white matter. Twenty-nine out of 71 patients (41%) who underwent spinal cord MRI showed intramedullary hyperintensity lesions on T2-weighted images. Twenty-four out of 51 patients (47%) who underwent enhanced spinal cord MRI had abnormal enhancement lesions. These were intramedullary lesions (12/51 (24%)) and meningeal lesions (13/51 (25%)). Images of brain and spinal cord MRI of representative patients are shown in Figure 1.

Eighty-five out of the 87 patients (98%) were treated with corticosteroids. The median period from symptom onset to the initiation of corticosteroids was 24 days (range: 7–1084). 

### 3.2. Types and Frequencies of Movement Disorders in GFAP-A Patients

The types and frequencies of movement disorders in GFAP-A patients are shown in Table 2. The most frequently observed movement disorder was ataxia (truncal and/or limb ataxia), and it was found in 43/87 patients (49%). The second most frequent movement disorder was tremor, observed in 39/87 patients (45%). Tremor was postural and/or an action tremor. The part of the body most affected by tremor was the upper limbs. Tremor in 21/39 patients (54%) was confirmed within 28 days. The third most frequent movement disorder was myoclonus, observed in 32/87 patients (37%). The parts of the body most affected by myoclonus were the upper and lower limbs. Myoclonus in 17/32 patients (53%) was confirmed within 28 days.

The least common movement disorders were dyskinesia, opsoclonus, rigidity, myokymia, and choreoathetosis (Table 2).

### 3.3. Characteristics of GFAP-A Patients with Movement Disorders

To examine the characteristics of the GFAP-A patients with movement disorders, we compared the demographics, clinical features, CSF characteristics, and neuroimaging findings from patients with and without movement disorders (Table 3). GFAP-A patients with movement disorders (median age, 54 years; range: 5–83 years) were significantly older than those without (median age, 30 years; range: 17–76 years) (*p* = 0.005). There were no significant differences in the other demographics between the two patient groups. There were also no significant differences between the two patient groups in terms of clinical findings, CSF characteristics, and neuroimaging findings. We examined the association between age and movement disorders using multivariable logistic regression analysis to control for the potentially confounding effects of gender and concomitant tumor. Older age was associated with movement disorders in GFAP-A (OR 1.052, CI: 1.011–1.094, *p* = 0.013).

### 3.4. Literature Review of Movement Disorders in GFAP-A Patients

In the previous literature, we found that 37 of 79 papers (47%) described movement disorders in GFAP-A patients (Table 4) [1,2,3,4,5,6,8,9,13,14,15,16,17,18,19,20,21,22,23,24,25,26,27,28,29,30,31,32,33,34,35,36,37,38,39,40,41]. In these reports, the most frequently described movement disorders were ataxia (23/79 (29%)), followed by tremor (22/79 (28%)), myoclonus (12/79 (15%)), and dyskinesia (6/79 (8%)). The least common movement disorders were hyperekplexia (3/79 (4%)), dystonia (2/79 (3%)), parkinsonism (2/79 (3%)), chorea (1/79 (1%)), axial stiffness (1/79 (1%)), grimacing (1/79 (1%)), oral movements (1/79 (1%)), catatonia (1/79 (1%)), and oculogyric crises (1/79 (1%)).

## 4. Discussion

In this study, we clarified the characteristics of movement disorders in individuals with autoimmune GFAP-A. The novel findings of this study are as follows. (1) Movement disorders are common neurological findings in GFAP-A patients. (2) The major types of movement disorders in this population were ataxia, tremor, and myoclonus. (3) GFAP-A patients with movement disorders were significantly older than those without.

The present study demonstrated that movement disorders are common in GFAP-A patients. In the previous literature, the most frequently described movement disorders were ataxia, followed by tremor and myoclonus. These three movement disorders were also common in our study population. Among patients with tremor and myoclonus, these symptoms were confirmed in about half of the patients within 28 days. These abnormal movements may thus be neurological findings characteristic of GFAP-A and can act as clinical features that would facilitate early diagnosis. 

The pathophysiology of movement disorders in GFAP-A patients is unclear. GFAP is expressed exclusively in astrocytes, including Bergmann glia in the cerebellum. Extensive inflammatory lesions can occur in the central nervous system of GFAP-A patients [1,2,8]. The observed movement disorders might have been complicated by various central nervous system lesions. Although abnormal intensities in the cerebellum on brain MRI are rare, ataxia in the GFAP-A patients might have been caused by cerebellar dysfunction [2,8]. Ataxia was usually mild and was observed in the early phase. Most patients had both limb and truncal ataxia. Although it improved after immunotherapy, truncal ataxia persisted in some patients. Tremor was predominantly postural and/or an action tremor in the upper limbs. A surface electromyogram from one patient showed an 11–12 Hz tremor, indicating an essential tremor. Structural changes in cerebellar Purkinje cells and neighboring neuronal populations have been observed in post-mortem studies of essential tremor, and GABAergic dysfunction and dysregulation of the cerebellar-thalamic-cortical circuitry have been documented [42,43,44]. Tremor may also be associated with impairment of the cerebellar pathways. Myoclonus in this population was usually non-segmental multifocal myoclonus in the upper and lower limbs. A previous report described a GFAP-A patient with multifocal myoclonus in whom hypermetabolism of the spinal cord was associated with areas of cortical hyper- and hypometabolism in 18F-fluorodeoxyglucose positron emission tomography imaging [25]. 

Dyskinesia, observed in this study, has previously been reported [4,5,6,17,23,41]. One of two patients with dyskinesia had an N-methyl-D-aspartate receptor (NMDAR)-IgG and ovarian teratoma. GFAP-IgG was sometimes accompanied by anti-neuronal antibodies, including NMDAR-IgG [2,3,13]. Dyskinesia is a common manifestation of NMDAR encephalitis [45,46]. If a GFAP-A patient is a young woman presenting with dyskinesia, examinations for NMDAR-IgG and ovarian teratoma should be performed because their presence is important for determining the treatment strategy.

In this study, two patients with no medical history of Parkinson’s disease or Parkinson-related disorders presented with rigidity in their upper limbs. The rigidity of one of these two patients was bilateral cogwheel rigidity dominant in the right upper limb. This patient also presented with bilateral hand postural tremor. The rigidity in these two patients improved after corticosteroid therapy. Brain MRI showed abnormal hyperintensity lesions, including in the cerebral white matter in both patients, and in the basal ganglia and the brainstem in one patient. Two reports have described parkinsonism in GFAP-A [6,14]. Tomczak et al. reported a case of GFAP-A with reversible parkinsonism, including rigidity, right hand action tremor, and bradykinesia [14]. This patient’s brain MRI showed nonspecific white matter changes and attenuated nigrosomes on susceptibility-weighted images. Although the pathophysiology of parkinsonism is unknown, GFAP-A patients may present with reversible parkinsonism.

We compared the demographics, clinical features, CSF characteristics, and neuroimaging findings from patients with and without movement disorders. The age of the GFAP-A patients with movement disorders was significantly higher than that of the GFAP-A patients without movement disorders. This result is important because movement disorders are common neurological features in elderly patients with neurodegenerative or cognitive disorders. GFAP-A should be differentiated in elderly patients with sudden-onset movement disorders.

The main limitations of this study are as follows: (i) the number of GFAP-A patients was small, (ii) the clinical information was based solely on the recall of the referring neurologist, (iii) the movement disorders were not evaluated by movement disorder experts, and (iv) the possibility of movement disorders becoming apparent during the clinical course cannot be ruled out in GFAP-A patients without movement disorders. To further examine the presence of movement disorders in GFAP-A patients, it will be necessary for movement disorder experts to prospectively observe clinical symptoms in a large group of GFAP-A patients. 

## 5. Conclusions

Movement disorders are a common neurological feature of GFAP-A. Ataxia, tremor, and myoclonus are the major movement disorders found in GFAP-A patients, and these may be clinical features that can facilitate early diagnosis. GFAP-A should be differentiated in elderly patients who present with sudden-onset movement disorders.

## Figures and Tables

**Table 1:** Demographics, clinical features, cerebrospinal fluid characteristics, and neuroimaging findings from 87 GFAP-A patients.

Variables	Patients (%)	Median (Range)
**Demographics**		
Age (N = 87)		51 (5–83)
Male	56/87 (64)	
Concomitant tumor	14/87 (16)	
Concomitant autoimmune disease	2/87 (2)	
Period from onset to admission (days) (N = 85)		12 (1–1054)
**Clinical findings**		
Movement disorder	74/87 (85)	
Urinary dysfunction	67/87 (77)	
Altered consciousness	66/87 (76)	
Nuchal rigidity and/or Kernig’s sign	56/87 (64)	
Fever	54/85 (64)	
Hyperreflexia	50/86 (58)	
Headache	43/85 (51)	
Cognitive dysfunction	42/87 (48)	
Psychosis	37/87 (43)	
Papillary edema	17/48 (43)	
Weakness	32/87 (37)	
Sensory disturbance	24/87 (28)	
Respiratory failure	19/86 (22)	
Convulsion	17/87 (20)	
**Cerebrospinal fluid findings**		
Cell counts (cells/μL) (N = 86)		79 (4–472)
Protein concentrations (mg/dL) (N = 86)		146 (30–320)
Oligoclonal IgG bands	43/61 (70)	
**Coexisting neural autoantibodies**		
Anti-MOG antibodies	2/57 (4)	
Anti-GAD antibodies	2/8 (25)	
Anti-NMDAR antibodies	2/42 (5)	
Anti-AQP4 antibodies	0/66 (0)	
**Brain MRI findings**		
T2/FLAIR hyperintensity lesions	76/86 (88)	
Hyperintensities in white matter	49/84 (58)	
Hyperintensities in basal ganglia	36/84 (43)	
Hyperintensities in cerebellum	5/84 (6)	
Gadolinium enhancement lesions	53/78 (68)	
LPRGE patterns	42/78 (54)	
**Spinal cord MRI findings**		
Intramedullary T2 hyperintensity lesions	29/71 (41)	
Gadolinium enhancement lesions	24/51 (47)	
Intramedullary enhancement lesions	12/51 (24)	
Meningeal enhancement lesions	13/51 (25)	
**Therapies**		
Corticosteroid therapies	85/87 (98)	
Period from onset to steroid initiation (day) (N = 83)		24 (7–1084)

AQP4: aquaporin 4, FLAIR: fluid-attenuated inversion recovery, GAD: glutamic acid decarboxylase, GFAP-A: autoimmune glial fibrillar acidic protein astrocytopathy, IgG: immunoglobulin G, LPRGE: linear perivascular radial gadolinium enhancement, MOG: myelin oligodendrocyte glycoprotein, MRI: magnetic resonance imaging. NMDA: N-methyl-D-aspartate.

**Table 2:** Types and frequencies of movement disorders in GFAP-A patients.

Movement Disorders	Patients (%)
Ataxia	43/87 (49)
Tremor	39/87 (45)
Myoclonus	32/87 (37)
Dyskinesia	2/87 (2)
Opsoclonus	2/87 (2)
Rigidity	2/87 (2)
Myokymia	1/87 (1)
Choreoathetosis	1/87 (1)

GFAP-A: autoimmune glial fibrillar acidic protein astrocytopathy.

**Table 3:** Demographics, clinical features, cerebrospinal fluid characteristics, and neuroimaging findings according to the presence or absence of movement disorders in GFAP-A patients.

Variables	With MDs		Without MDs		*p*
	Patients (%)	Median (Range)	Patients (%)	Median (Range)	
**Demographics**					
Age		54 (5–83), N = 74		30 (17–76), N = 13	0.005
Male	50/74 (68)		6/13 (46)		0.208
Concomitant tumor	11/74 (15)		3/12 (25)		0.400
Concomitant autoimmune disease	2/74 (3)		0/13 (0)		1.000
Period from onset to admission (days)		12 (1–1054), N = 74		8 (1–39), N = 13	0.139
**Clinical features**					
Urinary disturbance	59/74 (80)		8/13 (62)		0.165
Altered consciousness	55/74 (74)		11/13 (85)		0.726
Nuchal rigidity and/or Kernig’s sign	46/74 (62)		10/13 (77)		0.364
Fever	44/72 (61)		10/13 (77)		0.358
Hyperreflexia	43/73 (59)		7/13 (54)		0.768
Cognitive disfunction	37/74 (50)		5/13 (38)		0.553
Headache	34/72 (47)		9/13 (69)		0.228
Psychosis	31/74 (42)		6/13 (46)		0.771
Papillary edema	16/35 (46)		1/5 (20)		0.373
Weakness	28/74 (38)		4/13 (31)		0.760
Sensory disturbance	22/74 (30)		2/13 (15)		0.501
Respiratory failure	14/73 (19)		5/13 (38)		0.150
Convulsion	14/74 (19)		3/10 (30)		0.712
**Cerebrospinal fluid**					
Cell counts (cells/μL)		79 (4–378), N = 73		74 (10–472), N = 13	0.109
Protein concentrations (mg/dL)		136 (54–320), N = 73		156 (30–241), N = 13	0.376
Oligoclonal IgG bands	39/56 (70)		4/5 (80)		1.000
**Coexisting neural autoantibodies**					
Anti-MOG antibodies	2/50 (4)		0/7 (0)		1.000
Anti-GAD antibodies	1/7 (14)		1/1 (100)		0.250
Anti-NMDAR antibodies	2/38 (5)		0/4 (0)		1.000
**Brain MRI**					
T2/FLAIR hyperintensity lesions	64/73 (88)		12/13 (92)		1.000
Hyperintensities in white matter	43/71 (61)		6/13 (46)		0.371
Hyperintensities in basal ganglia	27/71 (38)		9/13 (70)		0.065
Hyperintensities in cerebellum	4/71 (6)		1/13 (8)		0.578
Gadolinium enhancement lesions	47/67 (70)		6/11 (55)		0.316
LPRGE	36/67 (54)		6/11 (55)		1.000
**Spinal cord MRI**					
Intramedullary T2 hyperintensity lesions	25/60 (42)		4/11 (36)		1.000
Gadolinium enhancement lesions	21/44 (48)		3/7 (45)		1.000
Intramedullary enhancement lesions	11/44 (25)		1/7 (14)		1.000
Meningeal enhancement lesions	11/44 (25)		2/7 (29)		1.000
**Therapies**					
Corticosteroid therapies	72/74 (97)		13/13 (100)		1.000
Period from onset to steroid initiation (days)	25 (7–1084), N = 70		14 (8–60), N = 13		0.136

FLAIR: fluid-attenuated inversion recovery, GAD: glutamic acid decarboxylase, GFAP-A: autoimmune glial fibrillar acidic protein astrocytopathy, IgG: immunoglobulin G, LPRGE: linear perivascular radial gadolinium enhancement, MDs: movement disorders, MOG: myelin oligodendrocyte glycoprotein, MRI: magnetic resonance imaging. NMDA: N-methyl-D-aspartate.

**Table 4:** Previous data on the presence of movement disorders in autoimmune GFAP astrocytopathy patients.

Movement Disorders	Number of Reports	References
Ataxia	23	[1,2,3,4,5,8,9,13,15,16,17,19,22,23,24,25,26,27,32,36,38,40,41]
Tremor	22	[1,2,6,8,13,14,20,21,24,26,27,28,29,30,31,33,34,35,36,37,38,39]
Myoclonus	12	[6,8,13,14,15,16,25,32,33,35,36,37]
Dyskinesia	6	[4,5,6,17,23,41]
Hyperekplexia	3	[6,32,35]
Dystonia	2	[6,23]
Parkinsonism	2	[6,14]
Chorea	1	[13]
Axial stiffness	1	[35]
Grimacing	1	[18]
Oral movements	1	[18]
Catatonia	1	[18]
Oculogyric crises	1	[35]

GFAP: glial fibrillary acidic protein.

## Data Availability

All data reported within the article are anonymized and available upon request from the qualified investigators.
